# A Review of Analytical Methods for Codeine Determination

**DOI:** 10.3390/molecules26040800

**Published:** 2021-02-04

**Authors:** Rimadani Pratiwi, Eka Noviana, Rizky Fauziati, Daniel Blascke Carrão, Firas Adinda Gandhi, Mutiara Aini Majid, Febrina Amelia Saputri

**Affiliations:** 1 Department of Pharmaceutical Analysis and Medicinal Chemistry, Faculty of Pharmacy, Universitas Padjadjaran, Bandung 45363, Indonesia; Rizky17007@mail.unpad.ac.id (R.F.); firas17001@mail.unpad.ac.id (F.A.G.); Mutiara17006@mail.unpad.ac.id (M.A.M.); febrina@unpad.ac.id (F.A.S.); 2 Department of Pharmaceutical Chemistry, Faculty of Pharmacy, Universitas Gadjah Mada, Yogyakarta 55281, Indonesia; eka.noviana@ugm.ac.id; 3 Departamento de Química, Faculdade de Filosofia, Ciências e Letras de Ribeirão Preto, Universidade de São Paulo, Ribeirão Preto 14040-901, Brazil; danielcarrao@yahoo.com

**Keywords:** codeine, drug analysis, colorimetry, spectrophotometry, electrochemistry, chromatography, capillary electromigration

## Abstract

Codeine is derived from morphine, an opioid analgesic, and has weaker analgesic and sedative effects than the parent molecule. This weak opioid is commonly used in combination with other drugs for over-the-counter cough relief medication. Due to the psychoactive properties of opioid drugs, the easily obtained codeine often becomes subject to misuse. Codeine misuse has emerged as a concerning public health issue due to its associated adverse effects such as headache, nausea, vomiting, and hemorrhage. Thus, it is very important to develop reliable analytical techniques to detect codeine for both quality control of pharmaceutical formulations and identifying drug misuse in the community. This review aims to provide critical outlooks on analytical methods applicable to the determination of codeine.

## 1. Introduction

Codeine (3-methylmorphine) is an alkaloid prepared from the methylation of morphine derived from poppy seeds (*Papaver somniferum*) [1]. It is used to manage mild to moderate pain, including chronic cancer pain, and has antitussive, antistress, and antidiarrheal properties [2,3,4]. In the body, a small amount of codeine is metabolized to form morphine (its active metabolite). Until now, the precise mechanism of action of codeine is not known; however, similar to morphine, codeine can bind to opioid receptors in the brain and induce signaling processes throughout the brain and the rest of the body. Codeine also has a sedative effect which helps reduce the pain sensation [1]. The drug has also been used in combination with acetaminophen or aspirin for more effective pain relief [1].

**Figure 1:**
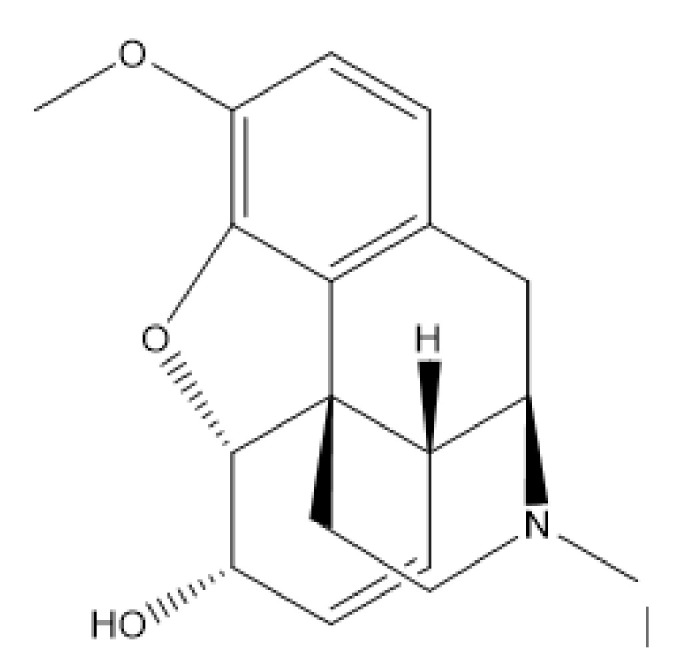
Structure of codeine.

The chemical structure of codeine is given in Figure 1. It has a benzene ring with heteroatoms bound to the ring which is capable of absorbing light energy in the ultraviolet (UV) range, allowing this compound to be analyzed by UV spectrophotometry [5]. There are various forms of codeine salts and hydrates including codeine phosphate, codeine hydrobromide, codeine N-oxide, codeine monohydrate, codeine phosphate sesquihydrate, codeine hydrochloride, and codeine phosphate hemihydrate [6]. Codeine monohydrate is a codeine base that has colorless crystals and is odorless, slightly soluble in water, and freely soluble in ethanol [7]. Codeine phosphate is often used for pharmaceutical formulations. It is a crystalline powder that is odorless, soluble in water, and slightly soluble in ethanol [7].

As codeine (especially its combination with non-opiate analgesics) can be easily obtained over the counter and has a psychoactive effect, it is often misused. Misuse of opioid analgesics including codeine has become an emerging public health concern due to its associated adverse effects on human health [8]. Reliable methods for detecting codeine in various samples are critical for both quality assurance of the drug within pharmaceutical formulations and identification of potential misuse in the community. Various methods have been reported for qualitative and quantitative analysis of codeine [1,9]. Qualitative or simple yes/no analysis often serves as a rapid test to detect the drug presence. This can be achieved via colorimetric assays which provide easy-to-interpret visual results [10]. Codeine can be quantified optically using spectrophotometric methods due to the presence of the chromophore group [11]. This drug is also electro-active, enabling electrochemical measurements for drug quantification [12]. However, the complexity of the matrices often poses a challenge to codeine detection, and thus rigorous sample preparation and/or coupling with separation techniques may be required to achieve accurate determination of the drug. This review aims to assess various analytical methods for codeine detection in the literature and provide critical outlooks on their advantages/disadvantages for analyzing codeine in different sample types. We selected seminal papers on the topic (regardless of the year of publication) to provide a broad overview on applicable methods, with examples (if applicable) provided by more recent publications (last 10 years). We start by evaluating simple colorimetric approaches for qualitative detection and then present two detection methods often used for codeine quantification: optical/spectrophotometric and electrochemical methods. Hyphenated detection methods with separation techniques including chromatography and electromigration-based separation are reviewed next before we finish with conclusions.

## 2. Colorimetric Assays

Colorimetric assays offer simple visual readouts and do not require complicated instruments for the analysis [10]. The United Nations International Drug Control Programme recommends several colorimetric assays for codeine detection which include the Marquis, Mecke, nitric acid, and ferric sulfate tests [13]. Froehde’s reagent can also be used to identify morphine and codeine. The Marquis and Mecke reagents give a violet color in the presence of opiates including morphine, codeine, and heroin [14]. The detailed color responses from various colorimetric reagents for opiates are shown in Table 1. Codeine reacts with Lieberman’s reagent, producing a black color; with Mandelin’s reagent, producing a green color; and it produces a violet color in the Marquis test [15]. Codeine is also identified by nitric acid reagent by producing an initial orange color that slowly changes to yellow [13]. Nitric acid and Mandelin’s reagents can be used to differentiate codeine from other opiates due to the noticeably different colors they produce for different opiates. Although these colorimetric methods are simple and rapid, most of the chromogenic reagents give similar color responses in the presence of different opioid drugs, making these techniques lack selectivity [16].

A rapid and selective colorimetric method for the detection of codeine sulfate has been developed using unmodified gold nanoprobes by Lodha et al. [17]. Citrate-stabilized gold nanoparticles (AuNPs) were synthesized and could react with codeine sulfate to produce a green color. The kinetics of AuNPs aggregation in the presence of codeine sulfate were obtained by measuring the absorbance and color intensity at the red, green, and blue channels as well as the total RGB. The results showed that there is a significant change in absorbance intensity from 520 to 582 nm upon increasing the concentration of codeine sulfate [17].

While limitations exist within the discussed colorimetric methods, they are still useful techniques for screening samples in large quantities, especially for those in resource-limited settings. In addition, it is possible to improve selectivity or even embedding specificity into the colorimetric approach by using specific recognition elements such as enzymes, antibodies, aptamers, or molecularly imprinted polymers [18,19,20,21]. However, as the assay complexity may increase with these specific recognition elements integrated, one should consider the cost-to-benefit ratio for the intended assay application.

## 3. Spectrophotometric Analysis

Ultraviolet/visible (UV/VIS) spectrophotometry is often employed in pharmaceutical analysis due to the simplicity of the measurement and the relatively low cost of the instruments [24]. Despite its simplicity, UV/VIS spectrophotometry is a well-established and powerful technique for analyzing single compounds such as bulk materials of active pharmaceutical ingredients (APIs) or excipients. A large UV/VIS spectra library of APIs is available as a reference to determine the identity and quantity of the analyzed sample [15]. The limitation of this technique, however, is the poor selectivity for detecting drug mixtures as compounds with similar chromophores will have overlapping absorption spectra [5]. To address this issue, derivative spectrophotometry and multivariate analysis have been implemented to resolve the overlapping bands and thus enable simultaneous detection of different drugs [25,26,27]. A list of spectrophotometric methods reported in the literature for codeine quantification is provided in Table 2.

Detection of codeine as a single compound in tablets using UV/VIS spectrophotometry was reported by Diaconu et al. [28]. The tablets were ground and 0.1 M NaOH was used to dissolve the drug. The absorbance of codeine was then measured at 284 nm wavelength. Another example was shown by Gharbavi et al., who measured codeine in water samples [29]. Due to the very dilute concentrations of codeine in the samples, they applied dispersive liquid–liquid microextraction preconcentration. The extracted samples were then measured at 270 nm, obtaining a detection limit of 18 μg/L for codeine.

Derivative spectrophotometry is an excellent technique for extracting both qualitative and quantitative information from spectra composed of unresolved bands [30]. The basic principle of this technique is the utilization of the first- or higher-order derivative of the absorbance spectra based on the wavelengths of the parent zero-order spectra [31]. The determination of the derivative values can be carried out by either graphic measurement, numeric measurement, or the zero-crossing technique [32]. Zero-crossing is a common technique in derivative spectrophotometry where the derivative spectrum crosses the zero point(s) of the *y*-axis at certain wavelengths. This is different from the numeric or graphic measurement where values determined numerically can be altered, yielding inaccurate results [25]. Edebi et al. used a zero-crossing technique to analyze codeine phosphate and diphenhydramine HCl in a cough mixture with zero-crossing points at 258 and 264 nm for codeine phosphate and diphenhydramine HCl, respectively [26]. Hoang et al. also used a similar technique for the simultaneous determination of paracetamol and codeine phosphate in tablets at zero-crossing points of 263.5 and 218.4 nm for the assay of paracetamol and codeine phosphate, respectively [33]. While spectra derivatization offers selective codeine detection in a drug mixture, the reproducibility of the technique highly depends on instrumental parameters such as the speed of the scan and the slit width and thus different instruments may provide different derivatization results [34]. In addition, there is a limit to the number of overlapped bands that the technique is capable of resolving. To date, only simultaneous detection of up to three compounds has been reported using derivatization spectrophotometry [25].

Multivariate analysis has also been implemented to simultaneously detect drug mixtures spectrophotometrically without separation. Dinç and co-workers analyzed a ternary mixture containing codeine phosphate, acetylsalicylic acid, and caffeine using inverse least-squares (ILS) and principal component regression (PCR) [27]. The ILS and PCR calibrations were constructed using a mixture of the three drugs. Excellent mean recoveries (i.e., 100.2% for ILS and 99.5% for PCR) and relative standard deviation (i.e., 1.23% for ILS and 2.10% for PCR) were obtained for codeine phosphate determination in the ternary mixture. The results of this study were also in agreement with results obtained by high-performance liquid chromatography and capillary electrophoresis.

Complete dissolution of the drug substance within the solvent is critical as the absorbance value is proportional to the concentration of the absorbing species in the dissolved form. In addition, particles from undissolved drugs or pharmaceutical excipients can scatter light and skew the measurements. Thus, the solvent must be selected based on the solubility of the analyzed drug. Dinç et al. dissolved ground tablets containing codeine phosphate, acetylsalicylic acid, and caffeine in 0.1 M HCl [27]. Since codeine and caffeine are weak bases, the acidic solution provides excellent dissolution of the drugs. However, for simultaneous detection of the three drugs, the solubility of acetylsalicylic acid should also be considered as the acidic drug will not dissolve well in an acidic aqueous solution. Duong and Fu used water and ethanol sequentially to dissolve codeine phosphate and paracetamol from ground tablets [33]. The sample was dissolved first in water, followed by a residue wash with ethanol.

Sample pre-treatment is typically needed to extract codeine from complex matrices such as biological samples prior to the analysis. Lotfi et al. developed a solid-phase extraction (SPE) technique using multi-walled carbon nanotubes for codeine analysis in human urine, achieving a detection limit of 0.4 μg/L [35]. Mashhadizadeh and Jafari used a cloud point extraction process with a non-ionic surfactant, Triton X-114, to extract codeine from blood samples [36]. Serum samples were collected from the whole blood and mixed with 1% Triton X-114 in the presence of acetate buffer pH 4.5 and bromothymol blue (BTB). The mixture was then centrifuged and cooled in an ice bath to separate the surfactant-rich phase containing codeine from the aqueous solution. The surfactant-rich phase has a distinguishable blue color due to the formation of an ion-pair complex between codeine and BTB and thus can be easily collected using a syringe pipette. A detection limit of 4.6 ng/mL was reported using this approach. Besides removing interfering species, extraction can also be applied for drug preconcentration, as shown by Gharbavi and coworkers previously via liquid–liquid microextraction. They used chloroform to extract codeine from water samples. The chloroform was then evaporated, and methanol was used to redissolve the drug. This technique provided a detection limit of 18 ng/mL [29].

Spectrophotometric methods are suitable for both analyzing a single drug and a mixture of drug compounds. The methods offer a relatively low-cost and easy implementation due to the wide availability of UV/VIS spectrophotometers in most laboratories. Sensitivity and selectivity are still considered as major limitations of these methods. However, sample extraction or preconcentration steps can be integrated to improve the detection limits, while the selectivity issue can be addressed using derivative spectrophotometry techniques or the application of multivariate data analysis.

## 4. Electrochemical Detection

The utilization of electrochemistry for detecting codeine in various types of samples has become a growing research interest in the past decades. Electrochemical detection offers improved sensitivity and selectivity over colorimetric detection and UV spectroscopy through the selection of electrode materials, detection schemes, and measurement techniques. Due to the miniaturization of electronic components needed for the measurements, electrochemical detection can also be carried out in the field using a portable instrument [38,39], increasing the applicability of the method for forensic and at-home clinical uses. In addition, electrochemical detection can be conveniently coupled with separation-based techniques such as liquid chromatography and capillary electrophoresis for multi-component analysis [40,41,42,43], and flow injection analysis (FIA) or batch injection analysis (BIA) for sample automation [42,44,45].

**Figure 2:**
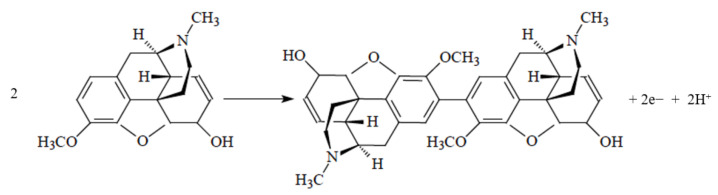
Proposed mechanism for the electro-oxidation of codeine [12].

Codeine is an electroactive species that can be oxidized via a proposed mechanism shown in Figure 2. Direct detection of codeine via this oxidation reaction has been applied in various methods reported in the literature [44,46,47]. Carbon electrodes such as carbon paste, glassy carbon, and boron-doped diamond (BDD) were mostly used for the detection (Table 3). Compared to metallic electrodes, the wider potential window of the carbon electrodes provides favorable electrochemical activity for many redox species [48], including codeine. This wider potential window is also desirable for the simultaneous determination of multiple analytes. For example, the determination of a mixture of codeine, acetaminophen, and caffeine has been demonstrated by Silva et al. using multi-walled carbon nanotubes (MWCNT) modified with a diamond-like carbon film [49]. The oxidation peaks were at 0.7, 1.2, and 1.5 V vs. Ag/AgCl for acetaminophen, codeine, and caffeine, respectively. In addition, carbon electrodes, especially composite materials, are often used in electrochemistry due to their biocompatibility, low cost, and easy fabrication [50]. While the conductivities of carbon electrodes are lower than those of metallic electrodes, various types of modifiers can be added to improve the electrochemical activity (Table 3). For example, by using carbon paste electrodes (CPEs) modified with Zn_2_SnO_4_ and graphene, Bagheri et al. were able to significantly improve the conductivity of electrodes from R_ct_ = 1400 in bare CPEs to only 160 Ω in Zn_2_SnO_4_/graphene-CPEs for the simultaneous detection of codeine and morphine [51].

While direct detection is the simplest strategy to quantify codeine, the relatively high potentials (often ≥ 1.0 V vs. Ag/AgCl) [46,49,52] needed for the direct oxidation of the analyte could provoke interference from other substances within the sample matrices. This problem can be overcome by the incorporation of a biorecognition element for the detection. For example, Bauer et al. reported the use of two enzymes: morphine dehydrogenase (MDH) and salicylate hydroxylase (SLH), on a Clark-type oxygen electrode [53]. Codeine was oxidized by MDH with a concomitant reduction of nicotinamide adenine dinucleotide phosphate (NADP^+^) into NADPH. The NADPH was then re-oxidized by SLH and the oxygen was consumed for the reaction elicited measurable current changes. Despite providing selectivity towards codeine, this approach cannot be used for codeine detection in the presence of related substances, for example, morphine, as MDH also oxidizes morphine. Asturias-Arribas et al. demonstrated two enzyme-based approaches for codeine detection. The first approach was based on the reversible inhibition of acetylcholinesterase (AChE) by codeine [54] and the second one utilized cytochrome P450 2D6 (CYP2D6) to oxidize codeine into morphine [55]. Similar to the previous enzymatic approach, these methods are also not specific towards codeine and thus caution should be taken if other AChE inhibitors or substrates for CYP2D6 are present in the sample matrices. Specific electrochemical detection of codeine has been reported by employing aptamers. For example, Saberian et al. immobilized a 5-hydroxy-1,4-naphthoquinone (Juglone)-labeled aptamer on a gold electrode [56]. Codeine binding to the immobilized aptamer induced a conformation change within the aptamer, which brought the redox label closer to the electrode surface, increasing the intensity of the electrochemical signal. Another aptamer-based sensor was proposed by Huang et al. for label-free detection of codeine [57]. A 37-mer aptamer sequence that had been optimized via the truncation–mutation assay was immobilized on a Au-mesoporous silica nanoparticle-modified glassy carbon electrode. The binding of codeine to the aptamer increased the electron transfer resistance and therefore reduced the measured signal. By combining the aptamer-based sensor with differential pulse voltammetry (DPV), a remarkably low detection limit (i.e., 3 pM) was achieved for codeine detection.

Amperometry is often applied for the detection of codeine in a flow-based system. Both single-potential and pulse amperometry have been coupled with either FIA or BIA for automated codeine determination [42,58,59]. Multiple-pulse amperometry (MPA) applies different pulses of potential sequentially and continuously to the working electrode as a function of time, which allows for both analyte detection and electrode cleaning between consecutive measurements. Compared to the commonly applied single-potential amperometry, MPA offers higher sensitivity, a lower detection limit from the negligible capacitive current, and less fouling to the electrodes [58,59]. Pulse voltammetric techniques such as DPV and square-wave voltammetry (SWV) are often used to improve detection limits for codeine quantification in stationary/quiescent solutions [46,47,57]. These pulse techniques rely on the difference in the rate of the decay of the charging (non-faradaic) and the faradaic currents following a potential step. Since the decay rate of the non-faradaic current is much faster than the faradaic current, the techniques can discriminate the faradaic current from the non-faradaic background. Nanomolar detection limits have been reported for codeine determination using DPV or SWV [51,60,61].

The complexity of sample preparation for electrochemical detection of codeine varies depending on the types of matrices. For solid samples such as pharmaceutical tablets, the preparation steps may include sample grinding and homogenization, followed by analyte dissolution into a suitable buffer or supporting electrolyte [47,52,55]. Undissolved materials from the matrices are then separated and removed via centrifugation and/or filtration. Centrifugation and filtration can also be applied to liquid samples that may contain undissolved components such as urine and blood serum/plasma to reduce interference from matrices during electrochemical measurements [52]. Direct measurement on diluted urine or serum samples without further separation has also been conducted by employing the standard addition method [47]. While the method minimizes matrix effects that interfere with measurement signals, the need for several spiked samples for analyte determination should be taken into account when choosing the method.

Electrochemical detection is a very versatile approach for codeine quantification. The sensitivity and selectivity of the assay can be tuned by carefully selecting the assay components (including the integration of biological recognition elements, enzymes for signal amplification), electrode materials, and measurement techniques. In addition, electrochemical detection can be performed using miniaturized/hand-held devices, similar to commercial glucometers, opening various possibilities to adapt the method for codeine determination at the point of care.

## 5. Chromatographic Analysis

Chromatographic analysis is a method of choice for detecting multi-drug components in a sample simultaneously. While spectral derivatization, multivariate analysis, and the use of specific recognition elements for multiplexed optical/electrochemical measurements have been successful to detect binary or ternary mixtures of drugs, none of these can beat chromatographic or separation-based detection techniques in term of the number of analytes they can resolve at the same time. In addition, the separation process allows for analytes stacking/preconcentrating into plugs or bands, which significantly improves the detection limit compared with direct detection methods. Various detectors such as optical detectors and mass spectrometry (MS) have been coupled to chromatographic systems to identify and quantify analytes. Table 4 provides a list of chromatographic methods that have been applied to codeine analysis.

### 5.1. Gas Chromatography-Mass Spectrometry (GC-MS)

Gas chromatography (GC) is a separation technique for volatile or semi-volatile constituents in the gas phase. GC can separate a mixture of compounds into individual components where each component can be detected by a detector. Nowadays, mass spectrometer is the most commonly used detector for GC [68], due to its applicability to detect various drug analytes based on their mass-to-charge ratio (*m*/*z*). GC-mass spectrometry (GC-MS) is sensitive and leads to definitive identification of codeine based on its characteristic MS fragmentation patterns [69]. This system offers high sensitivity for the detection and quantification of codeine, resulting in an LOD and LOQ in ng/mL [70,71].

Analysis using GC requires relatively complex derivatization steps. As the method can only work with volatile and thermally stable compounds, derivatizations are often needed to make analytes more GC-amenable. For example, N,O-bis(trimethylsilyl)trifluoroacetamide (BSTFA) is frequently used to derivatize labile groups such as hydroxyl on the target analytes with the more stable trimethylsilyl group [72]. A combination of BSTFA and 1% trimethylchlorosilane (TMCS) was reported by Rana et al. for derivatizing codeine, morphine, hydrocodone, and hydromorphone [71].

Lin et al. derivatized codeine and morphine with N-methyl-N-trimethylsilyltrifluoroacetamide (MSTFA) prior to sample injection to the GC system [73]. Before derivatization, they also performed acid/enzyme hydrolysis and solid-phase extraction of the drugs from urine samples. Another study reported extraction from urine samples using ethyl acetate, followed by derivatization with propionic anhydride [70]. A study by Kushnir et al. showed that GC derivatization using propionic anhydride provides more accurate and sensitive determination of analytes, compared with derivatizations using BSTFA, N-methyl-bis(trifluoroacetamide) (MBTFA), or heptafluorobutyric acid anhydride (HFAA) [74].

Besides urine samples, GC-MS has also been employed to analyze codeine in sweat. Sweat testing is less invasive than testing with blood or urine and therefore is more desirable for monitoring drug exposure in patients. For instance, Huestis et al. collected sweat specimens from patients using a sweat patch, and then drugs were extracted from the patch with sodium acetate buffer pH 4.0, followed by solid-phase extraction [75]. Extracts were later derivatized using BSTFA and 1% TMCS and analyzed by GC-MS simultaneously for determination of cocaine, codeine, and their metabolites.

### 5.2. High-Performance Liquid Chromatography (HPLC)

High-performance liquid chromatography (HPLC) is routinely used in laboratories for drug quantification in pharmaceutical dosage forms and clinical samples. Compared to GC, HPLC can accommodate a wider array of analytes including those that are non-volatile and thermally labile. Thus, complicated analyte derivatization steps are not necessary for HPLC analysis. HPLC has been reported to simultaneously detect several opioid drugs with similar structures such as heroin, morphine, and codeine in the sample due to its ability to separate the drugs [73]. Various detectors have been coupled with HPLC including optical detectors (e.g., photomultiplier tube (PMT) and photodiode array (PDA)) [76,77] and mass spectrometry (MS) [78,79,80,81,82].

The selection of stationary/mobile phase and separation conditions in HPLC highly depends on the physicochemical properties of the analytes and the complexity of the analyzed mixture [69]. Reversed-phase HPLC (RP-HPLC) systems with C-18 or C-8 columns have been reported for the determination of codeine/codeine phosphate and other pharmaceutical active ingredients in dosage forms [83,84,85]. In addition, these systems have also been used to detect codeine and its active metabolites in biological samples [86,87]. C-18 columns are popular in RP-HPLC systems due to their relatively high organic/carbon contents, allowing for better interaction between the organic solutes/analytes with the stationary phase [88]. These columns are also more stable at very low or very high pHs compared with columns with shorter alkyl chains [89,90].

Isocratic elution using a mixture of buffered solution and water-miscible organic solvents is often used for the determination of codeine in combination with other drugs. For example, Somsmorn et al. [76] performed isocratic elution for quantitative analysis of codeine and caffeine in a kratom (*Mitragyna speciosa* Korth.) cocktail. The mobile phase (0.01 M KH_2_PO_4_/methanol/acetonitrile/isopropanol (74:8:9:9, *v*/*v*/*v*/*v*)) was flowed at 1.0 mL/min in an Eclipse XDB-C8 column, resulting in a baseline separation (R_s_ = 1.5) of codeine and caffeine within 5 min. Prior to the chromatographic analysis, the kratom cocktail was concentrated by a vacuum freeze drier, followed by reconstitution of the dried sample in the mobile-phase solvents. For analysis in pharmaceutical dosage forms, samples are typically diluted with the eluents (for liquid formulations) or dissolved in the eluents followed by filtration to remove undissolved matters (for solid formulations). For instance, Maslarska et al. used an eluent mixture of acetonitrile and buffered solution (pH = 2.5) at 15:85 to dissolve codeine and paracetamol from tablet samples [83]. In addition to the isocratic elution, a gradient in eluent composition can be implemented to maximize analyte separation while keeping the analysis time to a minimum. For example, Hood and Cheung used a gradient combination of mobile phase A (methanol/glacial acetic acid/triethylamine (980:15:6 *v*/*v*)) and mobile phase B (water/glacial acetic acid/triethylamine (980:15:6 *v*/*v*)) for the simultaneous analysis of codeine phosphate, ephedrine HCl, and chlorpheniramine maleate in a cough–cold syrup formulation [91]. The elution was achieved in less than 7 min at a 1.5 mL/min flow rate.

Besides the conventional aqueous organic mixtures, micellar media can also be used as alternative eluents in RP-HPLC. This approach is often referred to as micellar liquid chromatography (MLC). In addition to their ability to facilitate separation of compounds with a wide range of polarities, micellar media are greener alternatives since they involve a lower quantity of organic modifiers and generate less toxic waste in comparison with the conventional organic eluents [92]. Belal et al. demonstrated MLC for the determination of paracetamol, caffeine, and codeine in tablets and human plasma [87]. They also assessed three different columns: C8, C18, and cyano columns, for the drug separation. Separation in the C8 column resulted in low resolution between caffeine and paracetamol, while the C18 column yielded long retention to codeine (i.e., 16.8 min retention time). The best system was obtained using the cyano column where the three analytes could be well resolved within 5 min.

HPLC with tandem MS or LC-MS/MS has also been increasingly popular in drug analysis. Several excellent review articles on LC-MS/MS applications for drug determination in clinical analysis and drug discovery have been published [93,94,95]. Thus, interested readers are encouraged to refer to these reviews for additional references. LC-MS/MS has recently become a gold standard for benzodiazepine determination [78] and many other drugs including codeine have been successfully quantified using this method. LC-MS/MS offers improved accuracy and precision and wide applicability to various analytes due to the universal nature of the MS detector. Hu et al. reported LC/MS-MS combined with liquid–liquid extraction for the simultaneous determination of codeine, ephedrine, guaiphenesin, and chlorpheniramine in beagle dog plasma, achieving a limit of quantification of 0.08 ng/mL [79]. Another sample preparation for LC/MS-MS analysis was reported using solid-phase extraction to extract codeine, morphine, 6-acetylmorphine, hydrocodone, hydromorphone, oxycodone, and oxymorphone from neat oral fluid [80]. Furthermore, Sproll et al. used a cold extraction method using methanol containing 0.1% acetic acid to extract opiate alkaloids from poppy seeds [81].

LC-MS/MS has emerged as the method of choice for drug abuse screening [96,97,98]. Mass spectra from MS analysis can be compared to a large database of reference drug spectra, allowing identification of the abused drugs [98]. Hei-Hwa Lee et al. successfully identified benzodiazepines and some new psychoactive substances in urine by LC/MS-MS via multiple reaction monitoring [99]. The samples were diluted in bicarbonate buffer pH 9.5, extracted with ethyl acetate, passed through a 0.22-μm polyvinylidene difluoride filter, and then injected into an ACE5 C18 column and eluted with a gradient of an acetonitrile/formic acid mixture. The retention time of codeine was 7.2 min and the Q1 mass was found at 300.1 *m*/*z*. Meanwhile, I-Lin Tsai et al. reported an ultra-high performance liquid chromatography-quadrupole time-of-flight mass spectrometry (UHPLC-QTOF-MS) method for screening 62 abused drugs and their metabolites in urine [100]. The samples were diluted 5-fold in deionized water, and then injected into a superficially porous micro-particulate C18 column and eluted with an acetic acid-based mobile phase. The retention time of codeine was 4.35 min with the parent ion found at 300.1 *m*/*z*.

HPLC offers various advantages for detecting codeine in drug combinations including selectivity, sensitivity, and wide applicability to various drugs (especially with an MS universal detector). The automation in HPLC analysis also greatly improves the reproducibility of the analysis and the ability to handle a large number of samples. Thus, this method is particularly of interest for routine analysis in pharmaceutical industries and clinical laboratories. The drawback of the method, however, is the high cost and relatively large size of the instrumentations, making HPLC not well-suited for applications in the field or at the point of care.

## 6. Capillary Electromigration Techniques

Capillary electromigration techniques (CETs), such as capillary electrophoresis (CE), have been used for the analysis of drugs of abuse and their metabolites [101]. CETs are separation techniques based on the resolution of analytes through differences in their migration mobility inside a capillary under the influence of an electric field [102]. Besides CE, other CETs such as non-aqueous capillary electrophoresis (NACE), micellar electrokinetic chromatography (MEKC), capillary electrochromatography (CEC), microemulsion electrokinetic chromatography (MEEKC), and capillary isotachophoresis (CITP) have been successfully employed for the analysis of drugs of abuse [101,103]. CETs are analytical tools that offer as main advantages high efficiency and resolution power, low reagent volumes, and sample consumption as well as lower costs (compared to chromatographic techniques), compatibility with several detection techniques, and automation [104]. The main limitation of CETs is their low sensitivity (mostly using UV/VIS detection), which is associated with the injection of low volumes of samples. However, several injection strategies have been used to enhance sensitivity and overcome this drawback [105,106].

Codeine (Figure 1) is a basic compound with a pKa value of 8.2 [11]. There are several methods in the literature reporting the analysis of codeine employing CETs (Table 5). In recent years, CE has been the main mode used for the determination of codeine using CETs [42,59,107,108,109,110,111,112,113,114,115,116,117,118,119,120]. Ciura et al. (2017) developed a CE analytical method for the analysis of codeine, arecoline, and papaverine, using 160 mM Tris and 200 mM phosphoric acid at pH 2.5 as the background electrolyte (BGE) and a separation potential of 30 kV. The authors evaluated sample injection precision and sensitivity with several injection modes. The obtained results were similar to those from HPLC analysis [115]. NACE and MEKC modes have been used as alternatives for the analysis of codeine [121,122,123,124]. Rodríguez et al. (2014) reported a NACE analytical method for the simultaneous analysis of codeine, morphine, imatinib, and its metabolite in urine. NACE is efficient for the analysis of hydrophobic compounds. The separation was performed using 15 mM ammonium acetate and 1% acetic acid in methanol as the BGE. The authors concluded the method was accurate, precise, sensitive, and specific for its application [121]. Anres et al. (2013) developed an MEKC analytical method, with 46 mM aqueous sodium phosphate buffer, pH 2.1/ACN 80:20 *v/v* containing 70 mM SDS (sodium dodecyl sulfate) as the BGE and a separation potential of -15 kV, for the analysis of codeine and five other compounds in urine. In MEKC, surfactants such as SDS are added to the BGE to form micelles (with opposite charges to the analytes). Field-enhanced sample injection coupled with sweeping and MEKC (FESI-sweep-MECK) was used, which allows the determination of traces of the analytes [124]. Although other CETs such as CEC, MEEKC, and CITP have the potential to be employed for codeine analysis, they have not been used recently and may be explored in the future.

Recently, CET methods have been used for the analysis of codeine in several samples such as pharmaceutical formulations [42,59,114,116], water [108], urine [109,117,119,121,122,123], saliva [120], exhaled breath [113], hair [107,118], liver microsomes [110], and plant extracts [111,112]. Sample preparation techniques are essential for the extraction of the analytes to perform clean-up of the sample and preconcentration of the analytes before analysis by CETs [125]. Liquid–liquid extraction [107,123], solid-phase extraction [121], dispersive micro solid-phase extraction [113], magnetic solid-phase extraction [109], pressurized liquid extraction [118], ultra-sonification extraction [111,112], and protein precipitation [110,120] have already been employed as sample preparation procedures for codeine analysis. Furthermore, in-line sample techniques have already been used in analysis employing CETs, in-line solid-phase extraction capillary electrophoresis (SPE-CE) [108,118,119], and in-line magnetic solid-phase extraction capillary electrophoresis (MSPE-CE) [117], which increases the possibility of automation of the method. Botello et al. (2012) employed an in-line solid-phase extraction of drugs of abuse, including codeine, for determination in water samples. The SPE extraction was performed using a small segment of the capillary filled with Oasis HLB sorbent, which was inserted in the fused silica capillary. The SPE-CE method presented an LOD of 200 ng/mL of codeine with a sensitivity enhancement factor from 2282. The authors reported that the method demonstrated applicability in tap and river water samples, which were directly analyzed without any off-line sample preparation [108].

Besides the determination of codeine, the analysis of its derivatives such as hydrocodeine, acetyl codeine, codeine-6-β-glucuronide, and other illicit drugs can be performed with CETs. Hydrocodeine is a synthetic derivative from codeine that is twice as strong. Botello et al. (2012) reported a CE method for the simultaneous analysis of codeine, hydrocodeine, and other opioids in human urine. The analytical method presented selectively for the analytes, an LOD of 0.20 ng/mL, and an analysis time of 17 min [119]. Acetylcodeine is an impurity found in street heroin, which is metabolized in codeine. Chen et al. (2011) developed a method by the combination of magnetic solid-phase extraction with CE for the monitoring of codeine, acetylcodeine, heroin, and five other illicit drugs in human urine. The reported method proved to be precise (2.8–12.4%), accurate (85.4–109.7%), and selective for the analysis of all analytes in less than 15 min [109]. Codeine-6-β-glucuronide is the major urinary metabolite of codeine in humans. Bonvin et al. (2014) developed a NACE method for the analysis of glucuronide metabolites of illicit drugs, including codeine-6-β-glucuronide, using minimal sample preparation through a “dilute and shoot” approach. This method presented an LOD of 0.5 µg/mL for the glucuronide metabolite of codeine with an analysis time of 15 min [122].

CETs have been coupled to several detectors such as ultraviolet (UV) [108,109,111,120,123], diode array detectors (DAD) [113,114,115,117,118,121,124], capacitively coupled contactless conductivity detectors (C^4^D) [42,59,116], electrochemiluminescence (ECL) [112], and mass spectrometry (MS) [107,110,119,122] for the analysis of codeine. Most methods used UV and DAD detection, which offers adequate selectivity and acceptable sensitivity in most cases. Cakir et al. (2019) developed a CE method for the analysis of codeine, amphetamine, and morphine in exhaled breath condensate using DAD detection. The separation was performed in a bare, fused silica capillary with 100 mM phosphoric acid/TEA as a background electrolyte (BGE) at pH 2.5 and including 20% (*v*/*v*) methanol and a separation potential of 20 kV. The detection was performed at 210 nm and the method proved to be selective and sensitive for its application with a limit of quantification (LOQ) of 30 ng/mL for codeine [113]. C^4^D is a powerful electrochemical detector that can be used as an alternative for the analysis of non-chromogenic compounds [126]. Cunha et al. (2017) reported a CE method using C^4^D for the analysis of codeine, orphenadrine, promethazine, scopolamine, tramadol, and paracetamol in pharmaceutical formulations. The authors used 20 mM β-alanine + 4 mM of NaOH + 4 mM NaCl at pH 9.6 as the BGE, which has adequate mobility and pH, allowing the simultaneous determination of cations and anions with this universal detector. The developed method presented an LOD of 15 µM for codeine and an analysis time of 3 min [116]. ECL detection is based on the emission of light from an electrochemical reaction, which increases selectivity and sensitivity [127]. Gao et al. (2006) developed a CE method for the analysis of codeine and other bioactive compounds in plant extracts using ECL detection. For that, the authors employed an ionic liquid (1-ethyl-3-methylimidazolium tetra-fluoroborate (EMImBF_4_)) in the BGE to perform the analysis. In this method, the LOD for codeine was 0.25 µM [112]. Finally, MS is a powerful detection technique that can be coupled to CETs, overcoming the main drawback of these separation techniques. In recent years, with the advance of CE-MS electrospray (ESI) interfaces, there are in the literature methods with single quadrupole [122], time-of-flight (TOF) [107], and ion trap [119] mass spectrometers for codeine analysis. Gottardo et al. (2012) employed a CE-TOF method for the analysis of drugs of forensic interest, including codeine, in human hair. The authors used as the BGE a non-volatile buffer, 50 mM ammonium phosphate at pH 6.5, and a sheath liquid composed of an isopropanol/water mixture (50:50 *v*/*v*) in the ESI interface. The method was successfully employed for the determination of illicit drugs in human hair, with an LOD of 0.002 ng/mg for codeine, which was possible due to the high selectivity and sensitivity of the TOF mass spectrometer [107].

CETs are an excellent alternative for the analysis of codeine, offering several advantages such as high efficiency, low sample and reagent volumes, low cost, several modes, and compatibility with several detectors. Although sensitivity may be a drawback for CETs, the use of mass spectrometry detectors and techniques that enhance the signal may overcome this issue. Therefore, these techniques present a huge potential of being used for the analysis of drugs of abuse, including codeine.

## 7. Conclusions

Codeine is an opioid analgesic that is usually available through prescriptions or over the counter in combination with other drugs. Reliable analytical techniques are critical for both quality control of the drug in pharmaceutical dosage forms and the detection of potential misuse by patients. While there is no single method that is well-suited for all analytical purposes, methods can be selected based on the required analytical figures of merit and practical considerations. For example, routine analysis of the drug in centralized laboratories will most likely rely on automated, instrumented techniques such as HPLC, GC, and CE with various detectors and direct detection methods coupled to flow injection analysis. These instrumental techniques provide excellent sensitivity, accuracy, and precision which are pivotal for quality control of pharmaceutical formulations and providing accurate diagnostics for patients. While these techniques cannot be easily brought outside the lab for point-of-care and field analysis, simpler techniques such as rapid colorimetric testing would suffice for the job. Challenges, however, still exist in creating simple, yet integrative platforms for analyzing codeine in various sample matrices. These platforms are also ideally capable of detecting multi-components/analytes and thus can provide more information for further analysis using more sophisticated instruments. In addition, continuous efforts for improving sample preparation steps and analytical operations in the instrumental techniques are also necessary to provide a more efficient, economic, and greener analysis of codeine.

## Figures and Tables

**Table 1:** Color responses of different chromogenic reagents for detecting codeine, heroin, and morphine.

Compound	Codeine	Heroin	Morphine	Ref.
Marquis	violet	violet	violet	[15]
Mecke	blue to green	blue to green	blue to green	[15,22]
Froehde	light green	n/a	violet to grey	[15,23]
Mandelin	green to blue	blue-grey	light grey	[15]
Lieberman	Black	n/a	black	[15]
Ferric sulfate	n/a	n/a	red	[13]
Nitric acid	orange slowly changing to yellow	yellow slowly changing to light green	orange rapidly changing to red then slowly to yellow	[13]
AuNPs	green	-	-	[17]

**Table 2:** Spectrophotometric methods for codeine quantification.

Analyte	Matrix	Method	Maximum Wavelength	LOD/LOQ	% RSD/Recovery	Ref.
Codeine	Tablet	UV/VIS spectrophotometry	284 nm 0.1 M NaOH	NA	RSD = 0.0003	[28]
Codeine	Water	UV/VIS spectrophotometry	270 nm in methanol	LOD = 18 ng/mL	RSD = 1.9% Recovery = 97.2–97.9%	[29]
Codeine	Human urine	UV spectrophotometry	265 nm in methanol	LOD = 0.4 ng/mL LOQ = 1.3 ng/mL	RSD = 1.56% for 0.01 mg/L	[35]
Codeine phosphate, Diphenhydramine HCl	Cough mixture	Zero-order derivative UV spectrophotometry	258 nm for codeine phosphate; 264 nm for diphenhydramine HCl (in HCl)	LOD = 1000 ng/mL LOQ = 50,000 ng/mL	RSD = 2.64% Recovery = 96.99–102.4%	[26]
Codeine phosphate, Paracetamol	Tablet	First-order derivative UV spectrophotometry	263.5 nm for paracetamol; 218.4 nm for codeine phosphate (in ethanol)	LOD = 260 ng/mL LOQ = 870 ng/mL	RSD = 0.36% Recovery = 99%	[33]
Codeine phosphate, Acetylsalicylic acid, Caffeine	Tablet	Spectrophotometric simultaneous analysis by inverse least-squares (ILS) and principal component regression (PCR) techniques (chemometric)	The absorbance values were measured at 15 points in the wavelength range 220–290 nm (in HCl)	NA	RSD = 1.23% for ILS 2.1% for PCR Recovery = 100.2% for ILS 99.5% for PCR	[27]
Codeine	Acetaminophen codeine tablet and blood	UV/VIS spectrophotometry	430 nm in ethanol	LOD = 4.6 ng/mL	RSD = 2.15%.	[36]
Codeine, Paracetamol	Tablets	UV spectrophotometric	243 nm for paracetamol; 278 nm for codeine (in H_2_O:ACN 90:10 *v*/*v*)	LOD = 50 ng/mL LOQ = 165 ng/mL	RSD = 0.81% Recovery = 100.53%	[37]

UV: ultraviolet; UV/VIS: ultraviolet/visible LOD: limit of detection; LOQ: limit of quantification; RSD: relative standard deviation.

**Table 3:** Various electrochemical methods for codeine determination reported in the past decade.

Analyte	Matrix	Method	Electrode	LOD	% RSD/Recovery	Ref.
Codeine	Tablet and urine	Chrono-amperometry	Tetrathiafulvalene/AChE- modified screen-printed carbon	LOD = 20,000 nM	RSD = 3.3% Recovery = 102 ± 10% (tablet) 101 ± 11% (urine)	[54]
	Tablet and urine	Chrono-amperometry	CYP2D6-modified screen-printed carbon	LOD = 4900 nM	RSD = 8.9% Recovery = 105 ± 7% (tablet) 108 ± 13% (urine)	[55]
	Tablet, urine, and serum	SWV	Nanodiamond/dihexadecyl phosphate-modified glassy carbon	LOD = 54.5 nM	RSD = 3.2% Recovery = 84–95% (tablet) 88-101% (urine) 93-100% (serum)	[46]
	Tablet and urine	DPV	BDD	LOD = 80 nM	RSD = 5% Recovery = 95–103% (tablet) In agreement with HPLC-PDA (urine)	[47]
	Standard solution	DPV	Au-mesoporous Si NPs/aptamer-modified glassy carbon	LOD = 0.003 nM	-	[57]
Codeine Acetaminophen	Tablet, urine, and serum	Amperometry and DPV	Pd NPs/porous Si microparticle-modified CNT paste	LOD = 200 nM (amperometry) LOD = 300 nM (DPV)	RSD = 4.9% Recovery = 97–105% (tablet) 96–104% (urine) 97–99% (serum)	[52]
	Tablet, urine and serum	MPA with FIA	Cathodically treated BDD	LOD = 35 nM	RSD = 3–4% Recovery = In agreement with HPLC (tablet) 102–107% (urine) 98–108% (serum)	[58]
	Serum	DPV	TiO_2_ NPs-modified carbon paste	LOD = 18 nM	RSD = 1.3% Recovery = 95–100%	[60]
	Tablet, urine, and serum	SWV	NiO NPs/carbon black/DHP-modified glassy carbon	LOD = 480 nM	RSD = 8.8% Recovery = In agreement with HPLC (tablet) 98–110% (urine) 97–108% (serum)	[62]
	Pharmaceutical formulations, plasma, and urine	SWV	Graphene/CoFe_2_O_4_ NPs-modified carbon paste	LOD = 11 nM	RSD = 4% Recovery = 98 ± 20% (tablet) 99 ± 2% (syrup) 98–102% (urine) 98–102% (plasma)	[61]
	Tablet, urine, and serum	SWV	Cathodically treated BDD	LOD = 14 nM	RSD = 4.2% Recovery = 76–98% (tablet) 98–100% (urine) 98–106% (serum)	[63]
Codeine Acetaminophen Ascorbic acid	Tablet, urine, and serum	SWV	ZnCrFeO_4_ NPs-modified MWCNT paste	LOD = 10 nM	RSD = 2.1% Recovery = 93–102% (tablet) 94–99% (syrup) 91–102% (urine) 96–102% (serum)	[64]
Codeine Acetaminophen Caffeine	Urine and serum	SWV	Diamond-like carbon film grown on vertically aligned MWCNT	LOD = 160 nM	RSD = <8% Recovery = 91–110% (urine) 85–98% (serum)	[49]
Codeine Morphine	Urine and serum	DPV	Pt NPs/porous Si flour-modified ionic liquid carbon paste	LOD = 20 nM	RSD = 5.7% Recovery = 104–107% (urine) 96–103% (serum)	[65]
	Pharmaceutical formulations, urine, and plasma	DPV	Zn_2_SnO_4_ NPs/graphene-modified carbon paste	LOD = 9 nM	RSD = 3.2% Recovery = 99 ± 3% (syrup) 99 ± 3% (injection) 98–101% (urine) 97–104% (plasma)	[51]
	Pharmaceutical formulations, urine and plasma	DPV	dsDNA/MWCNT/PDDA-modified pencil graphite	LOD = 41 ng/mL	RSD = 6.9% Recovery = 102 ± 9% (syrup) 105 ± 14% (injection) 104 ± 10% (urine) 102 ± 10% (plasma)	[66]
Codeine Diclofenac	Tablet	Amperometry with BIA	BDD	LOD = 1000 nM	RSD = 0.9% Recovery = 99–104%	[42]
Codeine Promethazine	Pharmaceutical formulation	MPA with BIA	BDD	LOD = 140 ng/mL	RSD = 7.9% Recovery = 96–98%	[59]
Codeine Oxycodone	Plasma and urine	DPV	CoFe_2_O_4_ NPs-modified carbon paste	LOD = 20 nM	RSD = 0.1% Recovery = 98–103% (urine) 98–102% (plasma)	[67]

BDD: boron-doped diamond; BIA: batch injection analysis; CNT: carbon nanotubes; DHP: dihexadecylphosphate; DPV: differential pulse voltammetry; FIA: flow injection analysis; LOD: limit of detection; LOQ: limit of quantification; MPA: multiple-pulse amperometry; MWCNT: multi-walled carbon nanotubes; PDDA: poly(diallyldimethylammonium chloride); SWV: square-wave voltammetry.

**Table 4:** Chromatographic system for codeine determination.

Analyte	Matrix	Method	Elution Type and Mobile Phase/Flow Rate	Column/Temperature	Detector	LOD/LOQ	% RSD/Recovery	Ref.
Morphine Codeine	Human urine	GC-MS	Helium as the carrier gas; 1.0 mL/min	HP-1MS column; temperature programming	MS-selected ion monitoring (SIM) mode	LLOQ = 25 ng/mL	RSD = 13%, Recovery = 87.2–108.5%.	[70]
Morphine Codeine Hydrocodone Hydromorphone	Human urine	GC-MS	Hydrogen as the carrier gas; 1.0 mL/min	GC column; temperature programming	MS-electron impact mode	LOD = 50 ng/mL LOQ = 100 ng/mL	RSD = 2.3% Recovery = 99.97%	[71]
Cocaine Codeine Metabolites	Sweat	GC-MS	Helium as the carrier gas	HP-1 fused silica capillary column; temperature programming	MS-selected ion monitoring (SIM) mode	LOD = 2.5 ng/patch LOQ = 2.5 ng/patch	RSD = 3.0% Recovery = 111.1%	[75]
Codeine	Human plasma	RP-HPLC	Acetonitrile and 5 mM ammonium phosphate dibasic (8:92, *v*/*v*) adjusted to pH 5.8 with phosphoric acid; 1.0 mL/min	Reversed-phased C8 column; ambient temperature	Fluorescence	LOD = 5 ng/mL LOQ = 10 ng/mL	RSD = 1.35–16.1% Recovery = 82.7–108%	[86]
Mitragynine Codeine Caffeine Chlorpheniramine Phenylephrine	Kratom cocktail (*Mitragyna speciosa* Korth.)	RP-HPLC	Isocratic elution 0.01 M KH_2_PO_4_/methanol/ Acetonitrile/isopropanol (74:8:9:9, *v*/*v*/*v*/*v*); 1.0 mL/min	Eclipse XDB-C8 column; 25 °C.	PDA	LOD = 5 ng/mL LOQ = 10 ng/mL	RSD = 2.495% Recovery = 98.96%	[76]
Codeine phosphate Chlorpheniramine maleate	Oral syrup	RP-HPLC	Isocratic elution; 1% o-phosphoric acid in water/acetonitrile/methanol (78:10:12); 1 mL/min	Phenomenex C18 column; 23 °C	UV/VIS	LOD = 2263 ng/mL LOQ = 6859 ng/mL	RSD = 0.23% Recovery = 99.01%	[84]
Codeine phosphate	Culture fluid of *Rhodococcus*	RP-HPLC	Phosphate buffer (pH 3.0) and acetonitrile (15:85); 1 mL/min	C18-modified silica gel; 40 °C	PDA	LOD = 200 ng/mL LOQ = 500 ng/mL	RSD = 0.35% Recovery = 97.92%	[77]
Caffeine Codeine Paracetamol	Tablets and human plasma	Micellar liquid chromatography	140 mM sodium dodecyl sulfate, 25 mM phosphate buffer, and 10% acetonitrile at pH = 3; 1 mL/min	Cyano column; 35 °C	UV	LOD = 54 ng/mL LOQ = 164 ng/mL	RSD = 0.803% Recovery = 99.51%	[87]
Codeine phosphate Triprolidine Hydrochloride Pseudophedrine hydrochloride	Liquid formulation	RP-HPLC	Methanol/acetate buffer/acetonitrile (85:5:10, *v*/*v*); 1.5 mL/min	C18 column	UV	LOD = 54 ng/mL LOQ = 164 ng/mL	RSD = 0.6% Recovery = 99.4%	[85]
Codeine phosphate Paracetamol	Tablet	RP-HPLC	Isocratic elution; acetonitrile/buffer solution (pH = 2.5) (15:85); 1 mL/min	LiChrospher^®^ RP-18 column	UV/VIS	LOD = 60 ng/mL LOQ = 600 ng/mL	RSD = 0.6% Recovery = 99.33–100.3%	[83]
Codeine phosphate Ephedrine HCl Chlorpheniramine maleate	Syrup	RP-HPLC	Mobile phase A consisted of methanol/glacial acetic acid/triethylamine (980:15:6 *v*/*v*) and mobile phase B was water/glacial acetic acid/triethylamine (980:15:6 *v*/*v*); 1.5 mL/min	Zorbax XDB C8 column; 30 °C	UV-diode array	Not evaluated	RSD = 0.08% Recovery = 99.87–100.96%	[91]
Morphine Codeine Thebaine Papaverine Noscapine	Pericarpium papaveris (*Papaver somniferum* L.) in hot pot broth	UPLC-QqQ-MS	Gradient elution; methanol (solvent B) and water (solvent A); 0.3 mL/min	Acquity BEH C18 column; 40 °C	MS in positive electrospray ionization with multiple reaction monitoring (MRM)	LOD = 40 ng/kg LOQ = 100 ng/kg	RSD = 16.9–20.5% Recovery = 78.9–124%	[82]
Codeine Guaiphenesin Chlorpheniramine Ephedrine	Beagle dog plasma	LC-MS/MS	Formic acid: 10 mM ammonium acetate/methanol (0.2:62:38, *v*/*v*); 0.2 mL/min	Phenomenex Luna C18 analytical column;	MS-selected reaction monitoring (SRM) mode	LLOQ = 0.08 ng/mL	RSD = 7% Recovery = 91%	[79]
Codeine Morphine 6-acetylmorphine Hydrocodone Hydromorphone Oxycodone Oxymorphone	Neat oral fluid	LC-MS/MS	Mobile phase was initially 95% 5 mM ammonium formate in water with 0.1% formic acid, decreased to 85% over 2 min then 5% over 1.5 min; 0.5 mL/min	Agilent Poroshell 120 SB-C18 column;	MS-positive electrospray ionization (ESI) mode	LOD = 0.04 ng/mL LLOQ 1.5 ng/mL	RSD = 3.7% Recovery = 99.3%	[80]
Codeine Morphine Hydrocodone Hydromorphone Oxycodone 6-Acetylmorphine	Urine, serum, plasma, whole blood, and meconium	LC-MS-MS	Isocratic acetonitrile and 2mM ammonium formate buffer at pH 3.0. (15%:85%)	0.525 mL/min	MS in positive electrospray ionization with multiple reaction monitoring (MRM)	LOD = 1 LLOQ = 2 ng/mL in urine, serum/plasma, and whole blood; ng/g in meconium	Recovery = 90.8% in urine and 50.4% in meconium	[78]
Morphine Codeine	Poppy seed	LC-MS/MS	Gradient program; mobile phase A (water, 20 mM ammonium hydrogen carbonate, adjusted with ammonia to pH 9) and mobile phase B (water/methanol 5:95 (*v*/*v*), 20 mM ammonium hydrogen carbonate, adjusted with ammonia to pH 9); 0.2 mL/min	Reversed-phase Phenomenex, RP 18 Gemini column; 40 °C	MS-positive electrospray ionization (ESI) mode	LOD = 300,000 ng/kg	Precision = 7.4-9.0% Accuracy = 9.8–17.6%	[81]

GC: gas chromatography; HPLC: high-performance liquid chromatography; LC: liquid chromatography; LOD: limit of detection; LLOQ: lower limits of quantification; LOQ: limit of quantification; MS: mass spectrometry; PDA: photo diode array; QqQ-MS: triple quadruple mass spectrometry; RP: reversed-phase; UPLC: ultra-performance liquid chromatography.

**Table 5:** Capillary electromigration techniques for analysis of codeine.

Analytes	Matrix	CET	Analysis Conditions	Analysis Time	LOD/LOQ	% RSD/Recovery	Ref.
Amphetamine Codeine Morphine	Exhaled breath condensate	CE-DAD	Bare fused silica capillary (41.5 cm effective length, 50 µm I.D.) 100 mM phosphoric acid/TEA at pH 2.5 including 20% (*v*/*v*) methanol 20 kV, 25 °C	15 min	LOQ = 30 ng/mL	RSD = 0.60–9.70% Recovery = −1.70–5.40%	[113]
Paracetamol Caffeine Codeine	Pharmaceutical dosage forms	CE-DAD	Uncoated fused silica capillary (47.5 cm effective length, 50 μm I.D.) 25 mM Na_2_HPO_4_ buffer containing 10% methanol at pH 8.5 27 kV, 25 °C	5 min	-	-	[114]
Arecoline Codeine Papaverine	Standard	CE-DAD	Uncoated fused silica capillary (60 cm total length, 50 μm I.D.) 160 mM Tris and 200 mM phosphoric acid at pH 2.5 30 kV, 25 °C	9 min	LOQ = 4 ng/mL	-	[115]
Codeine Orphenadrine Promethazine Scopolamine Tramadol Paracetamol	Pharmaceutical formulations	CE-C^4^D	Fused silica capillary (40 cm effective length, 50 µm I.D.) 20 mM β-alanine + 4 mM of NaOH + 4 mM NaCl at pH 9.6 25 kV, 25 °C	3 min	LOD = 15,000 nM	Recovery = 94–104%	[116]
Cocaine Codeine Methadone Morphine	Urine	MSPE-CE-DAD	Fused silica capillary (71.5 cm effective length, 50 μm I.D.) with trapped magnetic particles Aqueous solution of 40mM ammonium acetate adjusted with 28% ammonium hydroxide to pH 8.7 15 kV, 25 °C	14 min	LOD = 2 ng/mL	RSD = 6.5–13.8%	[117]
Cocaine Benzoylecgonine 6-acetylmorphine Codeine Morphine Methadone	Hair	SPE-CE-DAD	Fused silica capillary (80 cm total length, 50 µm I.D.) 11 mM α-cyclodextrin in an aqueous solution of 80 mM sodium phosphate at pH 2.530 kV, 25 °C	32 min	LOD = 0.12 ng/mg	RSD = 2.48-9.70% Recovery = 87.85–95.32%	[118]
Imatinib Metabolite (Imatinib) Codeine Morphine	Urine	NACE-DAD	Fused silica capillary (29 cm effective length, 75 µm I.D.) 15 mM ammonium acetate and 1% acetic acid in methanol 22 kV, 25 °C	9 min	LOD = 40 ng/mL	Recovery = 80.3–102.2%	[121]
Morphine-3-β-glucuronide Codeine-6-β-glucuronide Naloxone-6-β-glucuronide Ethyl-β-glucuronide	Standard and urine	NACE-MS	Fused silica capillary (100 cm effective length, 50 µm I.D.) or uncoated fused silica capillary with a porous tip (100 cm effective length, 30 µm I.D.) 5 mM ammonium acetate in ACN-MeOH 60:40 (*v*/*v*) 30 kV	15 min	LOD = 500 ng/mL	-	[122]
Promethazine Codeine	Pharmaceutical formulation	CE-C^4^D	Fused silica capillary (10 cm effective length, 50 µm I.D.) 10 mM oxalic acid and 1.8 mM triethanolamine at pH 8.425 kV	0.5 min	LOD = 28,000 ng/mL	RSD = 3.9–4.7%	[59]
Quinine Propranolol Strychnine Atropine Nicotine Codeine	Standard	MECK-DAD	Fused silica capillary (51.5 cm effective length, 50 µm I.D.) 46 mM aqueous sodium phosphate buffer, pH 2.1/ACN 80:20 *v/v* containing 70 mM SDS−15 kV	--	-	-	[124]
Codeine Morphine Methamphetamine Ketamine Alprazolam Clonazepam Diazepam Flunitrazepam Nitrazepam Oxazepam	Urine	MECK-UV	Uncoated fused silica capillary (40.2 cm effective length, 50 µm I.D.) 50 mM NaH_2_PO_4_ buffer at pH 2.3 containing 10% methanol and 150 mM SDS −15 kV, 25 °C	18 min	LOD = 28,000 ng/mL	Recovery = 77.6%	[123]
Codeine Diclofenac	Pharmaceutical formulation	CE-C^4^D	Fused silica capillary (10 cm effective length, 50 µm I.D.) 10 mM Tris/Taps at pH 8.225 kV	1 min	LOD = 21,000 nM	RSD = 0.7–1.5%	[42]
2-ethylidene-1,5-dimethyl- 3,3-diphenylpyrrolidine Codeine Hydrocodeine 6-acetylmorphine	Urine	SPE-CE-MS	Fused silica capillary (91.5 cm effective length, 50 µm I.D.) 60 mM ammonium acetate at pH 3.8 30 kV	17 min	LOD = 0.20 ng/mL	RSD = 4.9%	[119]
Morphine Codeine 6-monoacetylmorphine	Saliva	CE-UV	Fused silica capillary (60.3 cm effective length, 75 µm I.D.) 100 mM phosphate buffer at pH 3) containing 20% methanol and 5% isopropanol (*v*/*v*) 25 kV	20 min	LOD = 7 ng/mL	RSD = 6.1–17.7%	[120]
2-ethylidene-1,5-dimethyl- 3,3-diphenylpyrrolidine Codeine Cocaine 6-acetylmorphine	Water	SPE-CE-UV	Fused silica capillary (53 cm effective length, 50 µm I.D.) 80 mM disodium phosphate anhydrous and 6 mM of HCl at pH 3 30 kV, 25 °C	7 min	LOD = 200 ng/mL	RSD = 3.2–7.6%	[108]
3,4-methylenedioxyamphetamine 3,4-methylenedioxymethamphetamine Methadone Cocaine Morphine Codeine 6-monoacetylmorphine	Hair	CE-MS	Uncoated fused silica capillary (100 cm total length, 50 µm I.D.) 50 mM ammonium phosphate at pH 6.5 15 kV, 20 °C	25 min	LOD = 0.002 ng/mg	-	[107]
Amphetamine Methamphetamine 3,4-methylenedioxyamphetamine 3,4-methylenedioxymethamphetamine Ketamine Codeine Acetylcodeine Heroin	Urine	CE-UV	Fused silica capillary (60 cm effective length, 75 µm I.D.) 30 mM PBS at pH 2.0 containing 15% *v/v* ACN 20 kV, 25 °C	15 min	LOD = 53 ng/mL	RSD = 2.8–12.4% Recovery = 85.4–109.7%	[109]
Amphetamine Ephedrine Methadone Pethidine Tetracaine Codeine Heroin	Liver microsomes	CE-MS	Fused silica capillary (70 cm effective length, 50 µm I.D.) 20 mM ammonium acetate at pH 9.022 kV, 25 °C	5 min	LOD = 1.0 ng/mL	RSD = 1.08–1.12%	[110]
Morphine Codeine Thebaine	Plant extracts	CE-UV	Fused silica capillary (50 cm effective length, 50 µm I.D.) 100 mM phosphate buffer pH 3.0 containing 5 mM α-cyclodextrin 20 kV, 25 °C	15 min	LOD = 2000 ng/mL	RSD = 1.6–2.9% Recovery = 2.7%	[111]
Hebaine Codeine Morphine Narcotine	Plant extracts	CE-ECL	Uncoated fused silica capillary (50 cm effective length, 25 µm I.D.) 25 mM borax and 8mM EMImBF_4_ at pH 9.1815 kV 5 mM Ru(bpy)3 and 50mM phosphate at pH 9.18 (detection cell)	6 min	LOD = 250 nM	RSD = 4.11–5.01%	[112]

C^4^D: capacitively coupled contactless conductivity detector; CE: capillary electrophoresis; CET: capillary electromigration techniques; DAD: diode array detector; ECL: electrochemiluminescence detector; I.D.: internal diameter; LOD: limit of detection; LOQ: limit of quantification; MSEP: magnetic solid-phase extraction, NACE: non-aqueous capillary electrophoresis, SEP: solid-phase extraction, TEA: triethanolamine; U: ultraviolet detector.
